# The Expression and Diagnostic Value of LncRNA H19 in the Blood of Patients with Osteoarthritis

**DOI:** 10.18502/ijph.v49i8.3893

**Published:** 2020-08

**Authors:** Liwu ZHOU, Yang WAN, Qiang CHENG, Ben SHI, Lei ZHANG, Shuo CHEN

**Affiliations:** Department of Orthopedics, Jinling Hospital, Nanjing University, Nanjing 210004, P.R. China

**Keywords:** Osteoarthritis, Diagnosis, Blood

## Abstract

**Background:**

To investigate the expression and diagnostic value of LncRNA H19 in the blood of patients with osteoarthritis.

**Methods:**

A total of 130 cases of patients with osteoarthritis admitted to Jinling Hospital, Nanjing, China from Jun 2016 to Jul 2017 were elected as the study group, and 100 patients who underwent physical examination in Jinling Hospital during the same period were selected as the control group. The differences in expression levels of LncRNA H19 between the two groups were compared, the diagnostic value of LncRNA H19 in osteoarthritis and its relationship with clinical characteristics of patients with osteoarthritis were analyzed.

**Results:**

The expression level of LncRNA H19 increased in peripheral blood of patients with osteoarthritis (*P*<0.05). The AUC, critical value, sensitivity and specificity of the diagnosis of osteoarthritis were 0.891, 1.879, 96.00% and 85.73%, respectively. The expression level of LncRNA H19 was related to K-L grading, and the expression level of LncRNA H19 increased with K-L grading. Pearson correlation analysis showed that LncRNA H19 was negatively correlated with bone metabolism indexes PINP, N-MID, BGP, BALP and Lysholm score (*P*<0.05), and positively correlated with bone metabolism indexes β-CTX, VAS score and WOMAC score (*P*<0.05).

**Conclusion:**

LncRNA H19 is highly expressed in peripheral blood of patients with osteoarthritis, which is closely related to the occurrence and development of osteoarthritis and has a good diagnostic value for osteoarthritis.

## Introduction

Osteoarthritis is the most common musculoskeletal disease in the world, characterized by joint cartilage degeneration, subchondral osteosclerosis and narrowing of joint space (1). According to the epidemiological statistics of osteoarthritis, the incidence of osteoarthritis in people over 65 yr old can reach 50% globally, and the incidence of osteoarthritis in people over 80 yr old is as high as 80% (2). Osteoarthritis is the second most important reason for patients to lose their ability to work after cardiovascular disease, which has caused serious impact on society and families (3,4). In 2003, osteoarthritis was the sixth leading cause of disability in the world, and it is expected to rise to the fourth leading cause of disability by 2020, causing serious impacts on society and families (4,5). Early diagnosis is of great significance to improve the prognosis of patients with advanced osteoarthritis for the treatment methods are very simple and there are no effective treatments except total joint replacement.

Currently, the gold standard for clinical diagnosis of osteoarthritis is X-ray combined with clinical symptoms, but X-ray is not sensitive to early diagnosis of osteoarthritis and often causes missed diagnosis (6). In recent years, some biomarkers related to osteoarthritis have been found, but only a few of them have clinical application value (7). Long non-coding RNAs (LncRNAs) are a class of non-coding RNAs with a length of more than 200 nucleotides which play a crucial role in translation, RNA splicing and gene regulation. They can regulate key processes such as cell proliferation, apoptosis and differentiation, and are considered as new biological markers of osteoarthritis (8, 9). LncRNA H19 can regulate the growth of chondrocytes and osteoblasts as well as the cell anabolism processes (10, 11), suggesting that LncRNA H19 may be involved in the development of osteoarthritis. However, there are few reports on the clinical significance of the expression changes of LncRNA H19 in osteoarthritis.

In this study, the expression of LncRNA H19 in the blood of patients with osteoarthritis was detected, and its clinical significance was analyzed to investigate the application value of LncRNA H19 in osteoarthritis.

## Methods

A total of 130 cases of patients with osteoarthritis admitted to Jinling Hospital, Nanjing, China from Jun2016 to Jul 2017 were elected as the study group, and 100 patients who underwent physical examination in the Hospital during the same period were selected as the control group, with age 25 to 65 yr old.

The inclusion criteria were that all patients in the study group met the diagnostic criteria for osteoarthritis of the American College of Rheumatology in 1994 (12), and all patients in the study group received X-ray radiological diagnosis and K-L radiographic grading (13). All patients in the control group had no previous history of joint disease and no obvious disease symptoms during the study period.

The exclusion criteria were that patients in the study group who had received treatment or in the terminal-stage, patient with other systemic inflammatory syndrome, patients with serious endocrine disorders or autoimmune diseases, pregnant women, patients with cardiovascular, liver and kidney lesions, nerve, mental illness, chronic pain syndrome, difficulty in language communication, joint bone tumors, all kinds of cancer and bone metastases recently with acute stroke were excluded.

This study was approved by the hospital Ethics Committee, and the informed consent was signed by patients or their families.

### qRT-PCR

The expression levels of LncRNA H19 in peripheral blood of the two groups were detected by qRT-PCR, the total RNA was extracted from lytic cells with TRIzol lysate (Thermo Fisher Scientific (China)), 15596018), procedures of extraction followed the instructions in the kit. The concentration and purity of the extracted RNA were analyzed by Evolution™ 201/220 ultraviolet spectrophotometer (Thermo Fisher Scientific (China)), and the A260/A280 values between 1.8 and 2.1 were considered to meet the experimental requirements. Three percent agarose gel electrophoresis (the gel electrophoresis kit was purchased from Shanghai jingke chemical technology Co., Ltd.) was used to analyze the integrity of RNA. EasyScript One-Step RT-PCR SuperMix kit was purchased from Beijing Transgen Biotech, RRNA Template 1 μg, Forward GSP (10 μM) 0.4 μl, Reverse GSP(10 μM) 0.4 μl, 2*One-Step Reaction Mix 10 μl, EasyScript One-Step Enzyme Mix 0.4 μl, rnrnase-free Water supplementary Reaction system value 20 μM, the reaction conditions were 40 °C 30 min, 94 °C 5 min, 94 °C 30 s, 60 °C 30 s, 72 °C 2 kb/min and 72 °C 10 min with a total of 40 cycles. The β-actin was used as the internal reference of the reaction, 2−ΔCt was used to analyze the results. Primer sequences were designed and synthesized by Thermo Fisher Scientific (China) (Table 1).

**Table 1:** Primer sequence

***Variable***	***Forward primer***	***Reverse primer***
LncRNA H19	5′- TGATGACGGGTGGAGGGGCTA -3′	5′- TGATGTCGCCCTGTCTGCACG-3′
β-actin	5′-CGAGGCCCCCCTGAAC-3′	5′-GCCAGAGGCGTACAGGGATA-3′

### Observational indexes

The expression levels of LnvRNA H19 of the two groups were compared; the diagnostic value of LncRNA H19 in osteoarthritis and its relationship with clinical features of patients with osteoarthritis were analyzed.

### Statistical analysis

SPSS19.0 (Asia Analytics Formerly SPSS China) was used. Enumeration data were expressed as (n(%)), chi-square test was used for comparison between two groups, measurement data were expressed as mean± standard deviation (mean±sd). Students’ t test was used for comparison between the two groups, Anova was used for comparison between multiple groups, and LSD test was used for back testing. Pearson test was used to analyze the correlation between the two groups of measurement data. ROC analysis was used to analyze the diagnostic value of LncRNA H19 in osteoarthritis. *P*<0.05 was statistically significant.

## Results

A total of 100 subjects were included in the control group, with 55 males and 45 females, aged (54.76±13.64) yr old. The patients were included in the study group, with 65 males and 38 females, aged (56.78±11.03) yr old. There were no statistical differences in gender ratio and comparison of age between the two groups. Other basic data of the study group are shown in Table 2.

**Table 2:** General Data

***Variable***	***Control group (n=100)***	***Research group (n=103)***	***χ^2^/t***	***P***
Gender (n(%))			1.380	0.240
Male	55 (55.00)	65 (63.11)		
Female	45 (45.00)	38 (36.89)		
Age (yr)	54.76±13.64	56.78±11.03	1.162	0.247
BMI (kg/m^2^)	23.53±3.06	24.00±4.81	0.828	0.409
Disease course (yr)		3.20±1.38		
K-L classification (n (%))				
Grade I		39 (37.86)		
Grade II		46 (44.66)		
Grade III		18 (18.07)		
VAS score		5.48±1.14		
Lysholm score		57.13±12.09		
WOMAC score		50.60±9.63		
Pathogenic site				
Hip joint		50 (48.54)		
Knee joint		38 (36.89)		
Others		15 (14.56)		
Red blood cell count (10^9^/L)		4.46±1.21		
White blood cell count (10^9^/L)		6.97±2.11		
Hemoglobin (g/L)		119.14±19.58		
PINP(ng/mL)		47.47±11.55		
β-CTX (ng/mL)		0.516±0.092		
N-MID(ng/mL)		21.41±4.71		
BGP (μg/L)		4.82±1.21		
BALP (U/L)		35.43±5.83		

Procollagen type I N-terminal peptide (PINP), N-terminal osteocalcin (N-MID), β-collagen I telopeptide (β-CTX), bone gla protein (BGP), bone alkaline phosphatase (BALP)

**Fig. 1:**
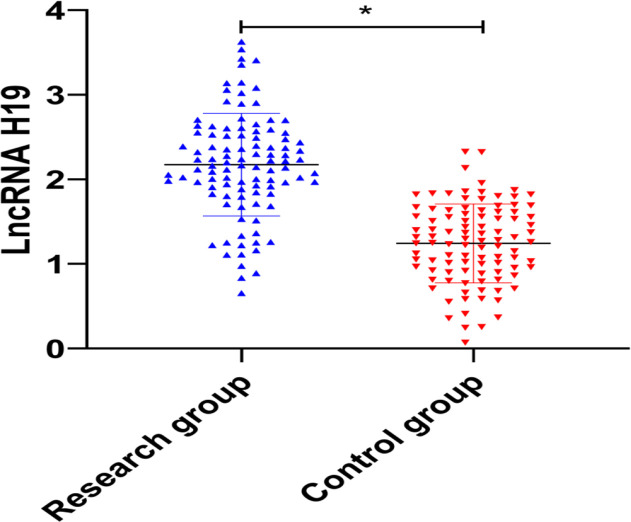
The expression level of LncRNA H19 in osteoarthritis The qRT-PCR results showed that the expression level of LncRNA H19 in peripheral blood of the study group was significantly higher than that of the control group. ^*^ means *P*<0.05

The expression level of LncRNA H19 in peripheral blood of the study group was 2.174±0.605, and that in peripheral blood of the control group was 1.243±0.466. The expression level in the study group was significantly higher than in the control group (*P*<0.05) (Fig. 1).

**Fig. 2:**
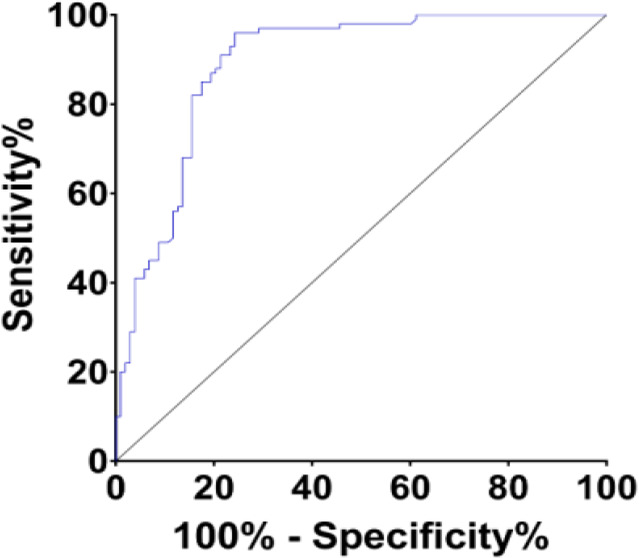
The diagnostic value of LncRNA H19 in osteoarthritis

The AUC, critical value, sensitivity and specificity of LncRNA H19 in peripheral blood in the diagnosis of osteoarthritis were 0.891, 1.879, 96.00% and 85.73%, respectively (Table 3, Fig. 2).

**Table 3:** The diagnostic value of LncRNA H19 in osteoarthritis

***Variable***	***LncRNA H19***
AUC	0.891
Critical value	1.879
95% confidence interval	0.8448±0.9366
Sensitivity	96.00%
Specificity	85.73%

**Fig. 3:**
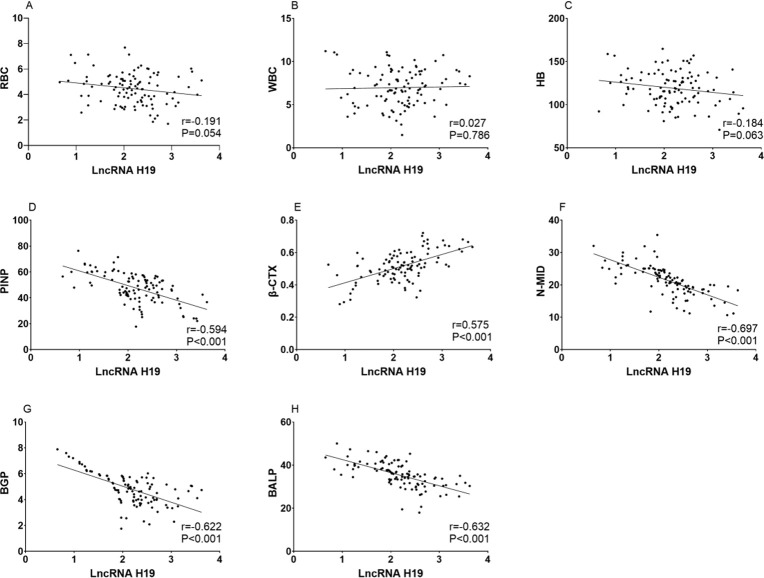
Relationship between LncRNA H19 and clinical features of patients with osteoarthritis A: The relationship between LncRNA H19 and red blood cell count in patients with osteoarthritis. B: The relationship between LncRNA H19 and white blood cell count in patients with osteoarthritis. C: The relationship between LncRNA H19 and hemoglobin level in patients with osteoarthritis. D: The relationship between LncRNA H19 and PINP level in patients with osteoarthritis. E: The relationship between LncRNA H19 and β-CTX levels in patients with osteoarthritis. F: The relationship between LncRNA H19 and N-MID level in patients with osteoarthritis. G: The relationship between LncRNA H19 and BGP level in patients with osteoarthritis. H: The relationship between LncRNA H19 and BALP level in patients with osteoarthritis

The expression level of LncRNA H19 was not correlated with gender, age, BMI, disease course and pathogenic site of osteoarthritis patients, but correlated with K-L grading. The expression level of LncRNA H19 increased with the increase of K-L grading. Pearson correlation analysis showed that LncRNA H19 was not correlated with red blood cell count, white blood cell count and hemoglobin level in patients with osteoarthritis, but negatively correlated with bone metabolism indexes PINP, N-MID, BGP and BALP (*P*<0.05) and positively correlated with bone metabolism indexes β-CTX (*P*<0.05) (Table 4, Fig. 3).

**Table 4:** Relationship between LncRNA H19 and clinical features of patients with osteoarthritis

***variable***	***n***	***LncRNA H19***	***t/F***	***P***
Gender (n(%))				
Male	65	2.196±0.635	0.518	0.606
Female	38	2.132±0.550		
Age (yr)				
<60	60	2.171±0.644	0.307	0.760
≥60	43	2.209±0.585		
BMI (kg/m^2^)			0.573	0.568
<24	53	2.144±0.637		
≥24	51	2.212±0.571		
Disease course (yr)				
<3	39	2.136±0.643	0.463	0.644
≥3	64	2.193±0.582		
K-L classification (n (%))				
Grade I	39	1.723±0.409	55.353	<0.001
Grade II	46	2.244±0.439^*^		
Grade III	18	2.973±0.400^*#^		
Pathogenic site				
Hip joint	50	2.109±0.617	1.369	0.259
Knee joint	38	2.301±0.633		
Others	15	2.067±0.455		

Notes:

* means *P*<0.05 compared with grade I, and

# means *P*<0.05 compared with grade II

The AUC, critical value, sensitivity and specificity of LncRNA H19 in peripheral blood in the diagnosis of osteoarthritis were 0.891, 1.879, 96.00% and 85.73%, respectively

**Fig. 4:**
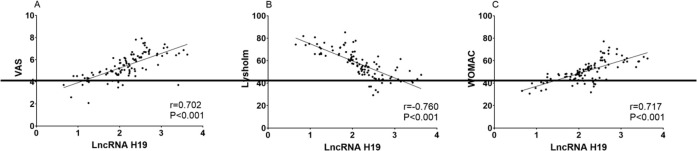
Correlation analysis between LncRNA H19 and disease severity in patients with osteoarthritis A: The relationship between LncRNA H19 and VAS scores in patients with osteoarthritis. B: The relationship between LncRNA H19 and Lysholm score in patients with osteoarthritis. C: The relationship between LncRNA H19 and WOMAC score in patients with osteoarthritis

Pearson correlation analysis results showed that LncRNA H19 was positively correlated with VAS score and WOMAC score of patients with osteoarthritis (*P*<0.05), and negatively correlated with Lysholm score (*P*<0.05) (Fig. 4).

## Discussion

The pathogenesis of osteoarthritis is complex, including genetic factors, physical factors, etc. The search for biomarkers related to osteoarthritis is of great significance to elucidate the pathogenesis of osteoarthritis, as well as the diagnosis and development of treatment methods for osteoarthritis (14, 15). LncRNAs are closely related to the development of bone and cartilage and can lead to cartilage extracellular matrix degradation (16).The diagnostic value of LncRNA H19 in osteoarthritis was analyzed, so as to find new indicators related to osteoarthritis and to provide auxiliary indicators for clinical diagnosis of osteoarthritis.

In recent years, more and more attention of researchers was paid on LncRNAs for its immune surveillance, cell cycle control, cell differentiation, embryonic stem cells maintain, and a variety of biological processes (17). LncRNA H19 had the effect of regulating the expression of collagen type II (18). Cartilage degeneration during osteoarthritis was associated with changes in chondrocyte metabolism, and the degradation of collagen type II could lead to defects in knee joint cartilage and promote the development of the occurrence of osteoarthritis (19).

The result of this study indicated that the expression of LncRNA H19 increased in peripheral blood of patients with osteoarthritis, suggesting that LncRNA H19 may be related to the occurrence of osteoarthritis. The ROC analysis showed that LncRNA H19 had a good diagnostic value for osteoarthritis, with an AUC of 0.891, sensitivity of 96.00% and specificity of 85.73% in diagnosing osteoarthritis.Furthermore, this study analyzed the relations between LncRNA H19 and clinical features of osteoarthritis patients, finding that there were no significant relations between LncRNA H19 and patients’ gender, age, BMI, course of diseases, and some routine blood index. However, LncRNA was closely related to bone metabolic index PINP, N-MID, BGP, BALP and β-CTX, suggesting that LncRNA H19 had good specificity and less interference factors in the diagnosis of osteoarthritis. And we also discovered that LncRNA H19 was associated with disease severity in patients with osteoarthritis. With the increase of K-L grading, the expression level of LncRNA H19 increased significantly, which was also correlated with VAS, WOMAC and Lysholm scores, further indicating that LncRNA H19 was closely related to the occurrence and development of osteoarthritis. The expression level of LncRNA H19 in osteoarthritic cartilage tissue was increased by microarray analysis with more than 4.57 times approximately (20). Combined with the study, it can be found that the differences in expression of LncRNA H19 in osteoarthritic cartilage tissue is more significant than that in peripheral blood, suggesting that LncRNA H19 in cartilage tissue may have better diagnostic value on osteoarthritis, but it needs further analysis. Other studies reported the LncRNA H19 mechanism of action in osteoarthritis. The help of LncRNA H19 in regulating anabolism and catabolism of cartilage cells may be related to its code of miR-675 (10). There were significant correlations between the expression levels of LncRNA H19, type II procollagen gene and mirR-675 in osteoarthritis cartilage tissue, and miR-675 could affect the expression of type II procollagen gene indirectly. MiR-141 could regulate the proliferation of osteoblasts by regulating miR-675, the target gene of LncRNA H19 and LncRNA H19 (11). We will further supplement the mechanism of LncRNA H19 in chondrocytes in future studies.

There were also some limitations in the study. This study explored only the diagnostic value of LncRNA H19 in osteoarthritis, the prognosis of LncRNA H19 in patients with osteoarthritis and the influence of treatment effect need further analysis. Because the regulating effects of LncRNA H19 on chondrocytes and osteoblasts, it may be as potential targets for osteoarthritis treatment. We will further complement the effects of LncRNA H19 may have on the treatment of osteoarthritis in the future study.

## Conclusion

LncRNA H19 is highly expressed in peripheral blood of patients with osteoarthritis, which is closely related to the occurrence and development of osteoarthritis and has a good diagnostic value for osteoarthritis.

## Ethical considerations

Ethical issues (Including plagiarism, informed consent, misconduct, data fabrication and/or falsification, double publication and/or submission, redundancy, etc.) have been completely observed by the authors.
